# 249. SARS-CoV-2 Spike Immune Complexes Induce NETosis in COVID-19

**DOI:** 10.1093/ofid/ofad500.322

**Published:** 2023-11-27

**Authors:** Kiana C Allen, Marcos J Ramos-Benitez, Heather Teague, Daniel S Chertow, Jeffrey R Strich, Seth Warner

**Affiliations:** Clinical Center, National Institutes of Health, Bethesda, Maryland; Clinical Center, NIH, Bethesda, Maryland; National Heart Lung and Blood Institute, National Institutes of Health, Bethesda, Maryland; Clinical Center, National Institutes of Health, Bethesda, Maryland; Clinical Center, National Institutes of Health, Bethesda, Maryland; Clinical Center, National Institutes of Health, Bethesda, Maryland

## Abstract

**Background:**

Disease severity in SARS-CoV-2 infection is associated with elevated levels of neutrophil extracellular traps (NETs) and antibodies. We previously demonstrated that plasma from patients with SARS-CoV-2 infection stimulates the release of NETs by healthy donor neutrophils and that this can be reversed by R406, a spleen tyrosine kinase (SYK) inhibitor. Immune complexes, stimulating through Fc receptors, induce robust NETosis through a SYK-mediated pathway. Therefore, we hypothesized that SARS-CoV-2 spike immune complexes can mediate the release of NETs from neutrophils and that this process can be reversed by R406.

**Methods:**

To test this, we created spike specific immune complexes utilizing an optimized concentration of affixed spike-trimer and SARS-CoV-2 patient plasma and evaluated the ability of these complexes to stimulate NETosis from healthy neutrophils. We quantified both IgG and IgA spike specific immune complex formation by ELISA utilizing HRP conjugated secondary antibodies (Promega Glomax Plate Reader). Healthy donor neutrophils were isolated, added to wells containing the spike immune complexes with and without R406, and live cell imaging (Incucyte S3) was used to visualize and quantify NETs.

**Results:**

Incubation of COVID-19 patient plasma with spike protein resulted in an average 7-fold and 9-fold increase in the formation of IgA and IgG spike specific immune complexes, respectively, compared to spike alone (Figure 1A). After stimulating healthy donor neutrophils with the spike immune complexes created from six SARS-CoV-2 patient plasma samples, we observed a mean 200% increase in the induction of NETs relative to spike alone (Figure 1B). There were positive correlations between immune complexes formation and NETs formed in cells exposed to those wells (IgA R^2^= 0.67, p=0.01 and IgG R^2^= 0.82, p=0.002). Preliminary data suggests that spike-immune complex NET formation can be reduced by R406 (n=3 COVID-19 plasma donors).

Figure 1A-B

**Figure:**
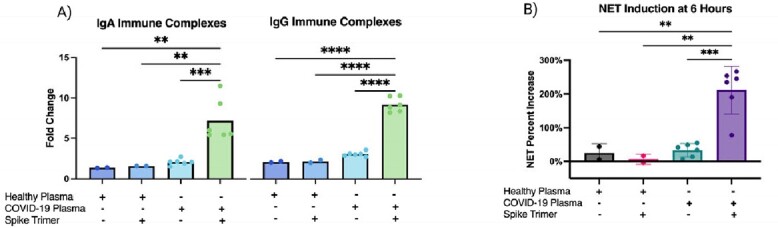

A) Quantification of IgA and IgG spike immune complexes was measured through measurement of absorbance at 405 nm after exposure to ABTS for 15 minutes. Value normalized to the spike alone condition ran on each day, and data represents a fold change from that condition (Ordinary One-way ANOVA: ** indicates p < 0.01; *** indicates p < 0.001; **** indicates p <0.0001). B) Induction of NETs 6 hours after exposure, demonstrating percent increase in NETosis in plasma isolated from COVID-19 patients (n=6) and healthy patients (n=2) in the presence and absence of SARS-CoV-2 spike immune complexes. Area of NETs was quantified by calculating the area (µm2 ) of ecDNA, visualized by Incucyte® Cytotox Green Dye. Area of NETs at T0 was subtracted from all conditions, and the induction of NETs was calculated as percentage increase relative to NETosis induced by SARS-CoV spike alone condition run on each day (Ordinary One-way ANOVA: ** indicates p < 0.01; *** indicates p < 0.001; **** indicates p <0.0001).

**Conclusion:**

Using our ELISA, we demonstrated that we can reliably generate spike-specific IgG and IgA immune complexes. Stimulation of healthy donor neutrophils by spike immune complexes increased NET-formation compared to spike alone providing evidence of immune complex mediated NET induction in SARS-CoV-2 infection.

**Disclosures:**

**All Authors**: No reported disclosures

